# The Ability of the Triglyceride-Glucose (TyG) Index and Modified TyG Indexes to Predict the Presence of Metabolic-Associated Fatty Liver Disease and Metabolic Syndrome in a Pediatric Population with Obesity

**DOI:** 10.3390/jcm14072341

**Published:** 2025-03-28

**Authors:** Sofia Tamini, Adele Bondesan, Diana Caroli, Nicoletta Marazzi, Alessandro Sartorio

**Affiliations:** Istituto Auxologico Italiano, Istituto di Ricovero e Cura a Carattere Scientifico (IRCCS), Experimental Laboratory for Auxo-Endocrinological Research, 28824 Piancavallo-Verbania, Italy; a.bondesan@auxologico.it (A.B.); d.caroli@auxologico.it (D.C.); n.marazzi@auxologico.it (N.M.); sartorio@auxologico.it (A.S.)

**Keywords:** obesity, pediatric obesity, children, adolescents, MASLD, MetS, triglyceride glucose index, TyG, TyG-body mass index, TyG-waist circumference index

## Abstract

**Background**: Metabolic-associated fatty liver disease (MASLD) and metabolic syndrome (MetS) are increasingly prevalent among children and adolescents with obesity, posing significant long-term metabolic and cardiovascular risks. Non-invasive identification of at-risk individuals is crucial for a timely intervention. This study aimed to evaluate the diagnostic performance of the triglyceride-glucose (TyG) index and its modified versions, TyG-body mass index (TyG-BMI) and TyG-waist circumference (TyG-WC), in predicting MASLD and MetS in a large cohort of children and adolescents with obesity. **Methods**: A total of 758 children and adolescents with obesity (454 females, 304 males; mean age 14.8 ± 2.1 years; mean BMI 37.9 ± 6.2 kg/m^2^) were included. MASLD was diagnosed via ultrasonography, while MetS was defined using International Diabetes Federation criteria. TyG, TyG-WC, and TyG-BMI were calculated for all participants. Receiver operating characteristic (ROC) curves were generated to assess the diagnostic accuracy of these indexes, including sensitivity, specificity, positive predictive value (PPV), and negative predictive value (NPV). **Results**: MASLD was detected in 38.9% of participants, with a higher prevalence in males (*p* < 0.0001). MetS was present in 27.8% of the cohort, with higher prevalence in males (*p* < 0.0001). Among the indexes, TyG-WC exhibited the highest sensitivity for MASLD (77.6%), whereas TyG-BMI had the highest specificity (63.3%). In predicting MetS, all three indexes performed better than for MASLD, with TyG demonstrating the highest PPV (54.5%) and TyG-BMI the highest NPV (87.5%). Predictive performance was lower in males than females, potentially due to sex-specific differences in fat distribution and metabolic response. **Conclusions**: TyG, TyG-WC, and TyG-BMI are promising, non-invasive tools for identifying children and adolescents with obesity at risk for MASLD and MetS. The superior sensitivity of TyG-WC and the high specificity of TyG-BMI highlight the value of incorporating anthropometric parameters into metabolic screening. Integrating these indexes into routine clinical practice may enhance early detection, allowing for timely intervention and personalized management strategies, ultimately reducing the long-term burden of metabolic and liver diseases in pediatric populations.

## 1. Introduction

Metabolic-associated fatty liver disease (MASLD) represents a growing public health concern in the pediatric population, particularly among children and adolescents with obesity. MASLD is the most common chronic liver disease in childhood [1,2]. It can be defined as a spectrum of conditions characterized by steatosis (i.e., the accumulation of fat in liver cells) in subjects who do not consume substantial amounts of alcohol [3]. If left untreated, MASLD can progress to non-alcoholic steatohepatitis (NASH), fibrosis, and even cirrhosis, with long-term consequences for liver health [3].

Numerous risk factors are associated with the onset and progression of MASLD, involving genetic and metabolic variables and lifestyle. Obesity, in terms of both general adiposity and excessive visceral fat, is one of the main risk factors for developing MASLD [1]. Additionally, metabolic syndrome (MetS) and insulin resistance are strongly correlated with the disease [1]. The prevalence of both MetS and MASLD is significantly higher among children and adolescents with obesity than in normal-weight subjects, and their coexistence poses significant long-term health risks [4]. MetS is defined as a group of concomitant metabolic alterations, including elevated blood pressure, hypertriglyceridemia, reduced high-density lipoprotein cholesterol (HDL-C), high fasting glucose levels, and central obesity (defined as increased waist circumference), all of which significantly increase the risk of type 2 diabetes and cardiovascular disease [5]. In this context, MASLD is often considered the hepatic manifestation of MetS [6,7,8,9].

Early identification of children and adolescents at high risk for these metabolic dysfunctions is essential for implementing preventive and therapeutic strategies. Timely intervention and targeted therapy can prevent the progression of the conditions to an irreversible state, improving liver function and reducing the persistence of MASLD and MetS into adulthood [10].

However, diagnosing MASLD remains challenging due to the limitations of current diagnostic tools [11,12]. Available diagnostic procedures for MASLD include clinical signs and symptoms, laboratory tests, radiological imaging, and a combination of clinical parameters with blood test results. While many of these markers are commonly used to assess patients with suspected MASLD, none demonstrate high enough specificity and sensitivity. Currently, liver histology remains the gold standard for diagnosing MASLD, as it is the only method capable of distinguishing simple steatosis or mild inflammation from NASH and accurately determining the presence and stage of fibrosis [13]. Performing liver biopsies in the pediatric population is challenging due to their invasive nature and should be avoided when possible. Thus, identifying new non-invasive diagnostic and screening alternatives is fundamental [14].

The triglyceride-glucose (TyG) index has emerged as a promising surrogate indicator for insulin resistance and metabolic dysfunction. Several studies have demonstrated its predictive value for MASLD and MetS in adults [15,16,17,18,19]. Moreover, modified TyG indexes, which incorporate anthropometric measures, such as body mass index (TyG-BMI) and waist circumference (TyG-WC), have been proposed to improve predictive accuracy [20,21,22,23]. These indexes integrate lipid and glucose parameters with anthropometric measures, offering a practical tool for assessing MASLD and MetS risk.

However, while these findings highlight the utility of TyG-based indexes in adults, their use in the pediatric population remains less explored. Given the increasing prevalence of MASLD and MetS, evaluating the effectiveness of these indexes in children and adolescents is essential for establishing their clinical relevance and developing new valuable and non-invasive methods of early screening and risk identification.

For these reasons, this study aimed to assess the ability of the TyG index, TyG-BMI, and TyG-WC to predict the presence of MASLD and MetS in a large cohort of children and adolescents with obesity.

## 2. Materials and Methods

### 2.1. Study Population

This retrospective study included children and adolescents with obesity who were hospitalized for a three-week multidisciplinary body weight reduction program (BWRP) at the Division of Auxology, Istituto Auxologico Italiano, IRCCS, Piancavallo-Verbania, between March 2018 and September 2022. The inclusion criteria were (i). individuals of both sexes, aged between 10 and 18 years, and (ii). body mass index (BMI) standard deviation score (SDS) > 2 according to the Italian reference growth charts [24]. The exclusion criteria were: (i). presence of genetic, endocrine, or iatrogenic forms of obesity, and (ii). absence of some of the data necessary for the index calculation.

All participants underwent a complete medical history review, physical examination, routine hematology and biochemistry screenings, and urine analysis. The study was approved by the Ethical Committee no. 5, Lombardy Region, Italy, Milan, Italy (ethical code number: 15/24, date of approval: 23 April 2024; acronym: TYGOBEPED). At admission to the hospital, all the patients and their parents had signed a written informed consent form for the anonymous use of all their clinical, anthropometric, and biochemical parameters for scientific purposes.

### 2.2. Anthropometry

Height and body weight (BW) were measured upon hospital admission following international guidelines [25] using a stadiometer scale (Wunder Sa.Bi., WU150, Trezzo sull’Adda, Italy), with the subject wearing only underclothing. BMI was calculated as:

- $\frac{\mathrm{weight}\;(\mathrm{kg})}{\mathrm{height}\;{\left(m\right)}^{2}}$.

Both hip and waist circumference (HC and WC, respectively) were measured with a flexible measuring tape. WC was measured at the midpoint between the last rib and the iliac crest while HC at the largest parts around the buttocks.

### 2.3. Laboratory and Clinical Parameters

Blood samples (about 10 mL) were collected early in the morning after an overnight fast in standard serum tubes. The same internal laboratory measured fasting plasma glucose (FPG), total (T-C), HDL (HDL-C), cholesterol, and triglyceride (TG) concentrations using standard methods.

Systolic (SBP) and diastolic (DBP) blood pressure were measured twice (3-min intervals in between) on the dominant arm with an aneroid sphygmomanometer (TemaCertus, Milan, Italy), by using appropriate-sized cuffs for young participants with obesity. The mean values were calculated and rounded to the nearest five mmHg value.

The presence of metabolic syndrome (MetS) was determined according to the IDF (International Diabetes Federation) criteria for diagnosis in children and adolescents [5]. In particular, MetS was defined in the presence of: WC ≥ 90th percentile for ages <16 years, and ≥94 cm for males and ≥80 cm for females aged >16 years, plus two or more of the following factors: (i). TG concentration: ≥150 mg/dL or in pharmacological treatment for dyslipidemia; (ii). HDL-C: <40 mg/dL for males and females aged <16 years, and <40 mg/dL for males and <50 mg/dL for females, or in pharmacological treatment for dyslipidemia; (iii). SBP ≥ 130 mmHg or DBP ≥ 85 mmHg; and (iv). fasting glycemia ≥100 mg/dL or diagnosis of type 2 diabetes mellitus.

The same expert echographist diagnosed MASLD through accurate liver ultrasonography using standard criteria [2,26,27].

### 2.4. Indexes

The three indexes were calculated according to the following formulas:

- TYG (males/females) [15,28] = $\ln(\frac{TG\;\left(\frac{\mathrm{mg}}{\mathrm{dL}}\right)\times FPG\;(\frac{\mathrm{mg}}{\mathrm{dL}})}{2}$);
- TYG-WC (males/females) [29] = TYG×WC;
- TYG-BMI (males/females) [29] = TYG×BMI.

### 2.5. Statistical Analysis

Continuous variables were expressed as the mean ± standard deviation, while categorical variables were presented as absolute and relative frequencies. Normality was verified using the Shapiro–Wilk test.

Receiver operating characteristic (ROC) curves were generated to determine the area under the curve (AUC), for each index as a predictor of MASLD and Mets. The optimal cutoff for each index was identified using the Youden index [30]. The analysis was performed in the entire study group and separately in the two genders.

The study group was divided into two subgroups based on the positive (MASLD+) or negative (MASLD−) diagnosis of MASLD and into females and males. Differences between groups (MASLD+ vs. MASLD− and females vs. males) were assessed using the *t*-student test for unpaired data or the Fisher’s exact test.

Furthermore, Pearson’s correlation was used to examine the association between the three indexes and the clinical and metabolic parameters considered. For the evaluation, r^2^ values below ±0.10 showed negligible correlation; values between ± 0.11 and ±0.39 weak correlation; values between ±0.40 and ±0.69 moderate correlation; values between ±0.70 and ±0.89 strong correlation and values above ±0.90 very strong correlation [31].

A *p* < 0.05 was considered statistically significant.

## 3. Results

The study included a total of 758 children and adolescents with obesity, 454 females and 304 males, with a mean age of 14.8 ± 2.1 years and a mean BMI of 37.9 ± 6.2 kg/m^2^.

MASLD was present in 295 patients (38.9%), while MetS was present in 211 patients (27.8%). Based on the presence or the absence of MASLD, the cohort was divided into the MASLD+ and the MASLD− subgroups, respectively. Table 1 shows the characteristics of the whole study population and of the MASLD+ and MASLD− subgroups.

Nearly all the parameters were significantly worse in the MASLD+ subgroup than in the MASLD− subgroup. Although both groups were comparable in terms of age, HC, and height, the MASLD+ group exhibited significantly higher BW (*p* < 0.0001) and BMI (*p* < 0.0001), greater WC (*p* < 0.0001), and higher levels of glucose (*p* < 0.01), T-C (*p* < 0.05), triglyceride (*p* < 0.0001), as well as increased SBP (*p* < 0.01) and DBP (*p* < 0.001). Additionally, HDL-C levels were significantly lower (*p* < 0.01). The values of all the indexes considered (TyG, TyG-WC, and TyG-BMI) were also higher in the MASLD+ subgroup than in the MASLD− subgroup (*p* < 0.0001 in each case).

The whole study group was divided based on gender into females and males. Table 2 shows the characteristics of the female and male groups.

No significant sex-related differences were observed in terms of age, HC, BMI, glycemia, and T-C. On the contrary, males exhibited significantly higher BW (*p* < 0.0001), WC (*p* < 0.0001), height (*p* < 0.0001), triglyceride levels (*p* < 0.01), SBP (*p* < 0.0001), and DBP (*p* < 0.01) and a greater prevalence of both MASLD and MetS (*p* < 0.0001 in both cases) compared to females. The values of all the indexes considered (TyG, TyG-WC, and TyG-BMI) were also higher in males than females. By contrast, females had higher HDL-C values (*p* < 0.0001) than males.

**Figure 1:**
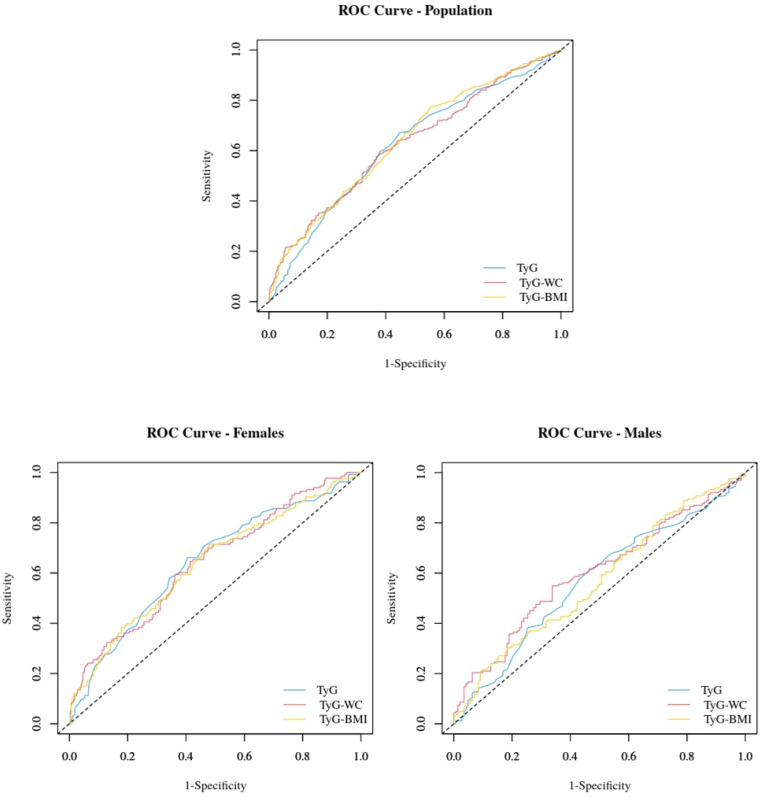
The receiver operating characteristic (ROC) curve compares the ability to predict the MASLD of TyG, TyG-WC, and TyG-BMI in the whole group and in the two genders.

Table 3 and Figure 1 show the ROC curve and the AUC values comparing the predicting ability of TyG, TyG-WC, and TyG-BMI for MASLD.

The three indexes were comparable in predicting MASLD in the three subgroups (*p* > 0.05 in all cases in the intragroup comparison); however, their performance was inferior in the male population.

In the whole study group, the ROC area of the TyG index was 0.62 (95% CI: 0.58–0.66), with an optimal cut-off of 4.43. This cut-off yielded a sensitivity of 67.1% and a specificity of 55.3%. The ROC area was slightly higher in the female population than in the whole study group (0.64; 95% CI: 0.59–0.70), with the same optimal cut-off (4.43) but slightly lower sensitivity (66.2%) and higher specificity (59.5%).

In males, the ROC area was the lowest (0.57; 95% CI: 0.50–0.63) compared to the whole study group and the female group, while the optimal cut-off was 4.47. In this case, the index’s sensitivity decreased to 61.1%, and the specificity decreased to 53.5%.

For the TyG-WC index, the ROC area in the whole study group was 0.64 (95% CI: 0.60–0.68), with an optimal cut-off of 478.83. leading to a sensitivity of 77.6% and a specificity of 44.5%. In females, the ROC area was 0.64 (95% CI: 0.58–0.69), with a similar optimal cut-off to that observed in the whole study group (478.80). However, with this cut-off, the index’s sensitivity was slightly lower (71.4%), while the specificity was higher (52.0%) than that observed in the whole study group. By contrast, the male subgroup exhibited the lowest ROC area (0.58; 95% CI: 0.50–0.63), with a markedly higher optimal cut-off (589.80). In this case, the index’s sensitivity was the lowest (30.2%), while the specificity was the highest (81.7%) compared to the whole study and female groups.

In the whole study group, the ROC area of the TyG-BMI index was 0.63 (95% CI: 0.59–0.67), with an optimal cut-off of 168.05, yielding a sensitivity of 57.6% and specificity of 63.3%. In females, the ROC area was 0.64 (95% CI: 0.59–0.70), with the same optimal cut-off (168.05), but slightly higher sensitivity (59.4%) and specificity (63.9%). In males, the ROC area (0.60; 95% CI: 0.54–0.66) was the lowest compared to the whole study and female groups, with an optimal cut-off of 170.38, leading to a sensitivity of 54.9% and specificity of 66.2%.

**Figure 2:**
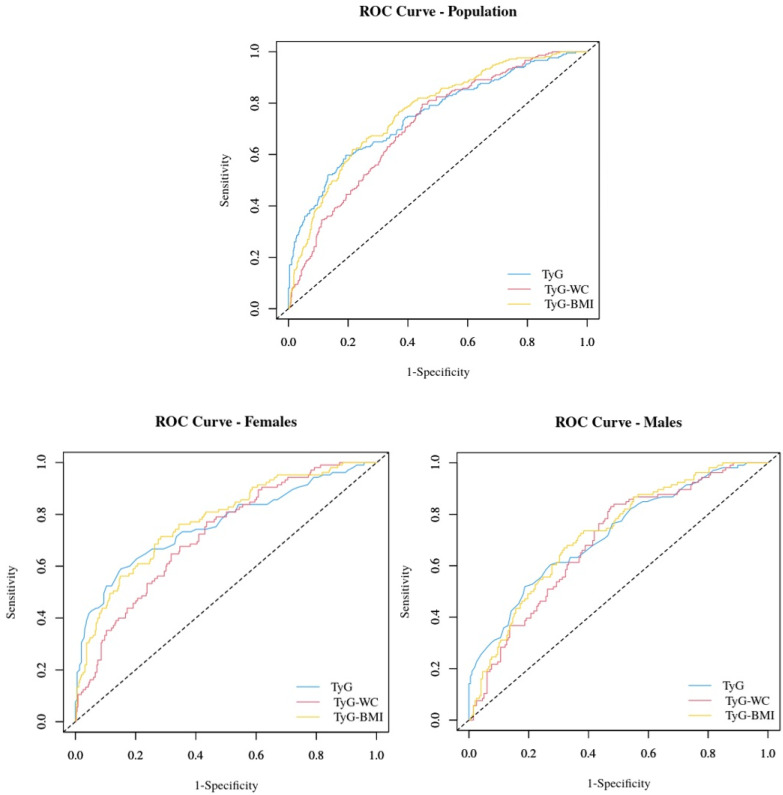
The receiver operating characteristic (ROC) curve comparing the ability to predict MetS of TyG, TyG-WC, and TyG-BMI in the whole group and in the two genders.

Table 4 and Figure 2 show the ROC curve and the AUC values comparing the predictive ability of TyG, TyG-WC, and TyG-BMI for MetS.

The three indexes performed better in predicting MetS than MASLD. Moreover, the indexes were comparable in predicting MetS in the three subgroups (*p* > 0.05 in all cases in the intragroup comparison).

In the whole study group, the ROC area of the TyG index was 0.75 (95% CI: 0.71–0.79), with an optimal cut-off of 4.55, yielding a sensitivity of 59.7% and specificity of 80.8%. In females, the ROC area was 0.76 (95% CI: 0.73–0.80), with the same optimal cut-off (4.55), which showed slightly lower sensitivity (59.0%) and higher specificity (84.8%). In males, the ROC area was slightly lower (0.72; 95% CI: 0.66–0.78), with an optimal cut-off of 4.59, resulting in reduced sensitivity (51.9%) and specificity (81.3%).

In the whole study group, the ROC area of the TyG-WC index was 0.76 (95% CI: 0.73–0.80), with an optimal cut-off of 538.48, leading to a sensitivity of 62.1% and specificity of 78.4%. In females, the ROC area was 0.77 (95% CI: 0.72–0.82), with a slightly lower optimal cut-off (510.40), higher sensitivity (71.4%), and slightly lower specificity (71.6%). In males, the ROC area was 0.72 (95% CI: 0.66–0.78), with an optimal cut-off of 531.10, resulting in the highest sensitivity (72.6%) but the lowest specificity (62.6%).

In the whole study group, the ROC area of the TyG-BMI index was 0.71 (95% CI: 0.67–0.75), with an optimal cut-off of 161.30, yielding a sensitivity of 79.6% and specificity of 55.2%. In females, the ROC area (0.72; 95% CI: 0.67–0.77) and the optimal cut-off (161.30) were similar to those observed in the whole study group. With this cut-off, the index’s sensitivity was slightly lower (77.1%), while the specificity was slightly higher (56.4%). In males, the ROC area was the lowest (0.69; 95% CI: 0.63–0.75), with an optimal cut-off of 159.87, resulting in the highest sensitivity (83.0%) but the lowest specificity (52.5%).

Table 5 shows the correlation between each index and some anthropometric and clinical characteristics in the whole study group and the population divided into males and females.

While some variables showed no significant correlation with the indexes, others exhibited varying degrees of association depending on sex and metabolic status.

In the whole population, the TYG index showed no correlation with age and HC, while it showed a weak correlation with height, BW, BMI, WC, SBP, DBP, and MASLD and a moderate correlation with MetS. In males, no correlation was observed between the TyG index and HC or MASLD, while there was a weak correlation with age, height, BW, BMI, WC, SBP, DBP and the presence of Mets. In the female population, TYG was not correlated with age, height, HC, or DBP. In contrast, it was weakly correlated with BW, BMI, WC, SBP, and the presence of MASLD, and it was moderately correlated with the presence of MetS.

The TYG-WC index in the entire population exhibited weak correlations with age, SBP, DBP, and MASLD, moderate correlations with height, HC, and MetS presence, and strong correlations with BW, BMI, and WC. In males, the index displayed weak correlations with SBP, DBP, and MASLD, moderate correlations with age, height, and SBP, and strong correlations with BW, BMI, HC, and WC. In females, weak correlations were observed with age, height, SBP, DBP, and MASLD, while moderate correlations with HC and strong correlations with BW, BMI, and WC were observed.

Lastly, in the whole study population, the TYG-BMI index exhibited weak correlations with age, height, SBP, DBP, and both MetS and MASLD presence, but strong correlations with BMI, BW, WC, and HC. In males, weak correlations were observed between height, DBP, and both MetS and MASLD presence, while moderate correlations with age and SBP and strong correlation with BMI and BW, WC, and HC were observed. In females, the TyG-BMI index showed no correlation with height, weak correlations with age, SBP, DBP and both MetS and MASLD presence, and strong correlations with BMI and BW, WC and HC.

## 4. Discussion

Pediatric obesity is an escalating public health challenge, with MASLD representing the primary hepatic manifestation of obesity and metabolic syndrome. As the most prevalent liver disorder among children and adolescents, MASLD carries significant long-term risks, including progression to NASH, fibrosis, and cirrhosis, with potential implications for liver transplant in adulthood. The increasing prevalence of pediatric MASLD parallels the alarming rise in childhood obesity rates, highlighting the urgent need for early identification and intervention [4,32].

Therefore, the aim of the present study was the evaluation of the diagnostic accuracy of three adiposity and metabolic dysfunction indexes (TyG, TyG-WC, and TyG-BMI) in predicting MASLD and MetS in a large cohort of children and adolescents with obesity.

In the study population, the prevalence of MASLD was 38.9%, with higher occurrence in males. This aligns with existing literature, which identifies male gender as a significant risk factor for MASLD, likely due to differences in body fat distribution, hormonal influences, and metabolic susceptibility [1,33]. Similarly, the prevalence of MetS in the cohort was 27.8%, reinforcing its strong association with MASLD and the broader metabolic dysfunction in pediatric obesity [4].

Nearly all the clinical and metabolic parameters were significantly worse in the MASLD+ subgroup than in the MASLD− subgroup. In fact, the subgroups showed comparable age, HC, and height. In contrast, the MASLD+ group showed significantly higher BW and BMI, greater WC, and higher levels of glucose, T-C, triglyceride, as well as increased SBP and DBP. Additionally, HDL-C levels were significantly lower. Also, the frequency of MetS was significantly higher in the MASLD+ subgroup.

When anthropometric and biochemical parameters were compared between the two genders, sex-based differences in metabolic risk were evident since males presented a worse clinical and metabolic profile than females. No significant gender-related differences regarding age, HC, BMI, and levels of glucose and T-C were found. However, WC, BW, height, triglyceride levels, SBP, and DBP were significantly higher compared in males compared to females. Moreover, females showed higher HDL-C values than males, which may offer some protective effect against metabolic dysfunction [34].

These characteristics align with previous studies indicating the sex dimorphism demonstrated in MASLD manifestations. In fact, in general, men have more risk factors and more severe pathophysiological outcomes than women [35,36].

Our analysis of the predictive performance of the three indexes suggests that the TyG index and its modified versions, TyG-BMI and TyG-WC, exhibit significant associations with MASLD and MetS in the analyzed pediatric population with obesity. These results agree with previous research in adult populations, where the TyG index has been recognized as a valuable marker for metabolic disturbances and insulin resistance [15,16,17,18,19]. The ability of these indexes to predict MASLD and MetS in children and adolescents, however, is less established, highlighting the importance of our study in expanding current knowledge.

When comparing the diagnostic performance of these indexes in the whole study population, TyG-WC demonstrated the highest performance, with the highest sensitivity for MASLD, while TyG-BMI showed the highest specificity. The general performance of the three indexes was comparable in the female population, but TyG-WC was still the index with the highest sensitivity and TyG-BMI was the one with the highest specificity. In the male population, the best performance was achieved by the TyG-BMI index, while TyG had the highest sensitivity and TyG-WC the highest specificity.

Notably, all three indexes performed better in predicting MetS than MASLD, and their individual performance was comparable in the entire study population and in the two genders. However, TyG-WC demonstrated the highest overall performance accuracy.

These findings suggest that incorporating waist circumference or BMI into the TyG index is helpful to enhance its predictive power, particularly in pediatric populations where body composition plays a crucial role in metabolic risk [24,27]. Similarly, the superior performance of TyG-WC can be attributed to the central role of abdominal obesity in metabolic dysfunction, emphasizing waist circumference as a critical marker for identifying children at higher risk [5,27].

Despite these findings, the predictive performance of the indexes remained moderate, particularly for MASLD. Recent studies have indicated that a multimodal approach combining metabolic indexes with imaging or biochemical markers provided superior predictive accuracy for MASLD [37,38]. In fact, the moderate AUC values obtained suggest that the TyG index, or its modified versions, alone may not be sufficient as a standalone diagnostic tool. Instead, its integration into a composite risk assessment model alongside other non-invasive markers, such as HOMA-IR, liver enzymes (ALT, AST), or inflammatory markers (CRP, IL-6) [39,40,41], could enhance the predictive reliability and improve risk stratification. Further research should focus on defining optimal cutoff values and also evaluating the cost-effectiveness of incorporating these indexes into routine clinical practice. Early identification of high-risk individuals through cost-effective strategies could help to optimize healthcare resources by targeting those subjects who would benefit most from further diagnostic testing and early interventions, thus potentially reducing long-term healthcare costs associated with MASLD and MetS.

When analyzing PPV and NPV of the indexes in the whole study group, we observed that their ability to correctly identify MASLD cases was moderate, with the highest PPV observed for TyG-BMI, while TyG-WC showed the highest NPV, suggesting that these two indexes have better reliability in identifying true positive or negative outcomes. This trend was similar in the sex-stratified analysis, with females exhibiting better negative predictive values than males, and males having greater positive predictive values than females.

For MetS, all three indexes demonstrated improved PPV and NPV compared to MASLD, reinforcing their stronger association with metabolic dysfunction. In general, TyG provided the best PPV, while TyG-BMI provided the best NPV. These findings highlight the importance of considering both sensitivity and specificity when selecting a diagnostic marker, as high specificity contributes to a higher PPV, while high sensitivity improves the NPV and reduces false-negative rates.

As already mentioned, these results suggest that the predictive ability of these indexes varies by sex, with males exhibiting lower overall accuracy than females.

This discrepancy may be attributed to differences in fat distribution, hormonal profiles, and metabolic responses between genders [35,36]. Previous studies have shown that males tend to accumulate more visceral fat, which is metabolically more active and contributes to insulin resistance and systemic inflammation [42,43,44]. Additionally, estrogens have been shown to exert hepatoprotective effects by modulating lipid metabolism, reducing hepatic steatosis, and improving insulin sensitivity [45,46]. In males, a deficiency of estrogens is associated with hepatic steatosis, as can be observed in the rare cases of inactivating mutations of the aromatase or of the estrogen receptor alpha. It has been demonstrated that many beneficial effects of estrogens are related to the activation of estrogen receptor alpha [47,48]. By contrast, androgens have been linked to the opposite effects, and a higher risk of metabolic dysfunction, further explaining the observed sex-based differences in MASLD prevalence and severity [46,47,49].

To the best of our knowledge, this is the first study to comprehensively assess the performance of TyG and its modified versions in predicting both MASLD and MetS in a large pediatric population with obesity. Our findings highlight the potential of these indexes as simple, non-invasive tools for early risk assessment in clinical settings. These indexes could be, in fact, used as first-line screening tools in primary care and pediatric endocrinology clinics to identify children at high risk for MASLD and MetS. Additionally, stratification models incorporating TyG-based indexes with other metabolic risk factors could refine patient selection for more intensive monitoring or targeted interventions, such as lifestyle modifications, pharmacological treatments, or advanced imaging for high-risk cases. Future research should focus on defining optimal cutoff values for clinical application and assessing their utility in real-world settings.

This study presents several strengths. First, the large sample size enhances the reliability and generalizability of our findings, allowing for a robust evaluation of the predictive ability of TyG and its modified indexes for MASLD and MetS in a pediatric population with obesity. Second, our study is among the first to systematically compare the performance of these indexes between males and females, providing valuable insights into sex-specific differences in metabolic risk. Third, using multiple indexes integrating metabolic and anthropometric parameters offers a more comprehensive assessment of MASLD and MetS risk. Lastly, including a well-characterized cohort, with detailed anthropometric, biochemical, and clinical evaluations, strengthens the methodological rigor of our study and ensures robust statistical analyses. As a limiting factor of the present study, the cross-sectional design prevents us from establishing causal relationships and causal inferences between TyG indexes and metabolic outcomes. In this respect, longitudinal studies are deemed necessary to confirm the predictive value of these indexes over time. Additionally, MASLD diagnosis was based on ultrasonography, which, although widely used, lacks the sensitivity of liver biopsy to detect the early stage of the disease, clearly distinguishing the degree of fat degeneration and assessing fibrosis severity [13,14]. Future research should explore the integration of transient elastography or MRI-based techniques for improving diagnostic accuracy. Finally, our study population consisted of hospitalized children undergoing a three-week multidisciplinary body weight reduction program, which may not fully represent the general pediatric population with obesity. Future research in community-based settings is necessary to improve the universality of our results.

In conclusion, the TyG index, TyG-BMI, and TyG-WC are promising simple, non-invasive markers for identifying children and adolescents at risk for MASLD and MetS. TyG-WC demonstrated the highest overall predictive ability among the three indexes, particularly in identifying MetS.

Given the rising prevalence of obesity-related metabolic disorders in pediatric populations, the integration of these indexes into routine clinical screening protocols may facilitate early diagnosis and timely intervention, ultimately reducing the long-term burden of metabolic and liver diseases. Further research is required to validate these findings, refine optimal cutoff values, and explore their applicability in clinical practice. Expanding research efforts in this area will contribute to improving early detection strategies and tailoring preventive measures for at-risk children and adolescents.

## Figures and Tables

**Table 1:** Main characteristics of the whole cohort and the groups with MASLD (MASLD+) and without MASLD (MASLD−).

	Total	MASLD+	MASLD−	*p*-Value
n.	758	295	463	
Sex (F/M)	454 (59.9%)/304 (40.1%)	133 (45.1%)/162 (54.9%)	321 (69.3%)/142 (30.7%)	<0.0001
Age (yrs)	14.8 ± 2.1	14.6 ± 2.3	14.8 ± 2.0	ns
Height (cm)	163.0 ± 9.8	163.4 ± 10.5	162.8 ± 9.3	ns
BW (kg)	101.6 ± 22.7	106.6 ± 26.7	98.4 ± 19.2	<0.0001
BMI (kg/m^2^)	37.9 ± 6.2	39.5 ± 7.1	36.9 ± 5.3	<0.0001
WC (cm)	115.2 ± 14.7	119.1 ± 15.8	112.8 ± 13.4	<0.0001
HC (cm)	121.6 ± 12.2	122.7 ± 14.0	121.0 ± 10.8	ns
SBP (mmHg)	125.5 ± 12.6	127.2 ± 13.5	124.4 ± 11.9	<0.01
DBP (mmHg)	78.5 ± 7.9	79.7 ± 8.3	77.8 ± 7.6	<0.001
Glucose (mg/dL)	81.5 ± 6.2	82.2 ± 6.5	81.0 ± 6.0	<0.01
T-C (mg/dL)	163.8 ± 31.7	166.6 ± 33.4	162.0 ± 30.2	<0.05
HDL-C (mg/dL)	42.8 ± 10.5	41.5 ± 9.8	43.6 ± 10.9	<0.01
Triglycerides (mg/dL)	96.6 ± 40.8	105.2 ± 42.5	91.1 ± 38.8	<0.0001
MetS (+/−)	211 (27.8%)/547 (72.2%)	100 (33.9%)/195 (66.1%)	111 (24%)/352 (76%)	<0.0001
TyG	4.4 ± 0.2	4.5 ± 0.2	4.4 ± 0.2	<0.0001
TyG-WC	512.7 ± 74.3	535.5 ± 79.0	498.2 ± 67.3	<0.0001
TyG-BMI	168.7 ± 30.1	177.6 ± 33.9	163.1 ± 25.8	<0.0001

Acronyms: BW, body weight; BMI, body mass index; WC, waist circumference; HC, hips circumference; SBP, systolic blood pressure; DBP, diastolic blood pressure; T-C, total cholesterol; HDL-C, HDL cholesterol; MetS, metabolic syndrome; MASLD, non-alcoholic fatty liver disease; TyG, triglyceride glucose index; ns, not significant.

**Table 2:** Characteristics of the whole study group divided into the two genders.

	Females	Males	*p*-Value
n.	454	304	
Age (yrs)	14.8 ± 2.1	14.6 ± 2.2	ns
Height (cm)	160.3 ± 7.4	167.0 ± 11.4	<0.0001
BW (kg)	97.2 ± 18.7	108.1 ± 26.4	<0.0001
BMI (kg/m^2^)	37.7 ± 6.0	38.3 ± 6.4	ns
WC (cm)	112.0 ± 13.5	120.1 ± 15.1	<0.0001
HC (cm)	122.3 ± 29.3	120.7 ± 13.2	ns
SBP (mmHg)	123.5 ± 12.1	128.4 ± 12.7	<0.0001
DBP (mmHg)	77.8 ± 7.6	79.7 ± 8.3	<0.01
Glucose (mg/dL)	81.2 ± 6.5	81.9 ± 5.7	ns
T-C (mg/dL)	162.8 ± 31.0	165.3 ± 32.4	ns
HDL-C (mg/dL)	44.3 ± 10.4	40.5 ± 10.3	<0.0001
Triglycerides (mg/dL)	93.1 ± 40.1	101.8 ± 41.5	<0.01
MetS (+/−)	105 (23.1%)/349 (76.9%)	106 (34.9%)/198 (65.1%)	<0.0001
MASLD (+/−)	133 (29.3%)/321 (70.7%)	162 (53.3%)/142 (46.7%)	<0.0001
TyG	4.4 ± 0.2	4.5 ± 0.2	<0.01
TyG-WC	495.9 ± 68.6	537.8 ± 75.5	<0.0001
TyG-BMI	166.9 ± 29.5	171.4 ± 30.7	<0.05

Acronyms: BW, body weight; BMI, body mass index; WC, waist circumference; HC, hips circumference; SBP, systolic blood pressure; DBP, diastolic blood pressure; T-C, total cholesterol; HDL-C, HDL cholesterol; MetS, metabolic syndrome; MASLD, non-alcoholic fatty liver disease; TyG, triglyceride glucose index; ns, not significant.

**Table 3:** ROC area, cutoff according to the Youden index, sensitivity, specificity, positive predictive value, negative predictive value, positive likelihood ratio, and negative likelihood ratio of the three indexes in predicting MASLD in the whole study group and the two genders.

	ROC Area	Cutoff	Sensitivity	Specificity	PPV	NPV	PLR	NLR
**Study group**								
TyG	0.62 (0.58–0.66)	4.43	67.1%	55.3%	48.9%	72.5%	1.50	0.60
TyG-WC	0.64 (0.60–0.68)	478.83	77.6%	44.5%	47.1%	75.7%	1.40	0.50
TyG-BMI	0.63 (0.59–0.67)	168.05	57.6%	63.3%	50.0%	70.1%	1.57	0.67
**Females**								
TyG	0.64 (0.59–0.70)	4.43	66.2%	59.5%	40.4%	80.9%	1.63	0.57
TyG-WC	0.64 (0.58–0.69)	478.80	71.4%	52.0%	38.2%	81.5%	1.49	0.55
TyG-BMI	0.64 (0.59–0.70)	168.05	59.4%	63.9%	40.5%	79.2%	1.65	0.64
**Males**								
TyG	0.57 (0.50–0.63)	4.47	61.1%	53.5%	60.0%	54.7%	1.32	0.73
TyG-WC	0.58 (0.50–0.63)	589.80	30.2%	81.7%	65.3%	50.7%	1.65	0.85
TyG-BMI	0.60 (0.54–0.66)	170.38	54.9%	66.2%	65.0%	56.3%	1.63	0.68

Abbreviations: TyG, triglyceride glucose index; MASLD, non-alcoholic fatty liver disease, PPV, positive predictive value; NPV, negative predictive values; PLR, positive likelihood ratio; NLR, negative likelihood ratio.

**Table 4:** ROC area, cutoff according to the Youden index, sensitivity, specificity, positive predictive value, negative predictive value, positive likelihood ratio, and negative likelihood ratio of the three indexes in predicting MetS in the whole study group and the two genders.

	ROC Area	Cutoff	Sensitivity	Specificity	PPV	NPV	PLR	NLR
**Study group**								
TyG	0.75 (0.71–0.79)	4.55	59.7%	80.8%	54.5%	83.9%	3.11	0.50
TyG-WC	0.76 (0.73–0.80)	538.48	62.1%	78.4%	52.6%	84.3%	2.88	0.48
TyG-BMI	0.71 (0.67–0.75)	161.30	79.6%	55.2%	40.7%	87.5%	1.78	0.37
**Females**								
TyG	0.76 (0.70–0.82)	4.55	59.0%	84.8%	53.9%	87.3%	3.89	0.48
TyG-WC	0.77 (0.72–0.82)	510.40	71.4%	71.6%	43.1%	89.3%	2.52	0.40
TyG-BMI	0.72 (0.67–0.77)	161.30	77.1%	56.4%	34.8%	89.1%	1.77	0.41
**Males**								
TyG	0.72 (0.66–0.78)	4.59	51.9%	81.3%	59.8%	75.9%	2.77	0.59
TyG-WC	0.72 (0.66–0.78)	531.10	72.6%	62.6%	51.0%	81.0%	1.94	0.44
TyG-BMI	0.69 (0.63–0.75)	159.87	83.0%	52.5%	48.4%	85.2%	1.75	0.32

Acronyms: TyG, triglyceride glucose index; MetS, metabolic syndrome; PPV, positive predictive value; NPV, negative predictive values; PLR, positive likelihood ratio; NLR, negative likelihood ratio.

**Table 5:** Correlation between each index and anthropometric and clinical characteristics in the whole study group and in the population divided into males and females.

		Age	Height	BW	BMI	WC	HC	SBP	DBP	MetS	MASLD
**Study group**											
TyG	R squared	0.066	0.111	0.196	0.183	0.218	0.089	0.167	0.116	0.407	0.187
	*p*-value	ns	<0.01	<0.0001	<0.0001	<0.0001	<0.05	ns	<0.01	<0.0001	ns
TyG-WC	R squared	0.334	0.458	0.799	0.744	0.950	0.673	0.394	0.343	0.4116	0.244
	*p*-value	<0.0001	<0.0001	<0.0001	<0.0001	<0.0001	<0.0001	<0.0001	<0.0001	<0.0001	<0.0001
TyG-BMI	R squared	0.306	0.235	0.837	0.967	0.772	0.808	0.395	0.345	0.317	0.235
	*p*-value	<0.001	<0.05	<0.0001	<0.05	ns	<0.0001	<0.0001	<0.0001	<0.0001	<0.0001
**Males**											
TyG	R squared	0.167	0.138	0.165	0.130	0.163	0.082	0.151	0.122	0.373	0.088
	*p*-value	<0.05	<0.05	<0.01	<0.05	<0.01	ns	<0.01	<0.05	<0.0001	ns
TyG-WC	R squared	0.558	0.563	0.860	0.812	0.946	0.790	0.431	0.332	0.354	0.130
	*p*-value	<0.001	ns	ns	<0.01	<0.001	<0.001	<0.0001	<0.0001	<0.0001	<0.05
TyG-BMI	R squared	0.469	0.356	0.855	0.967	0.848	0.845	0.468	0.383	0.293	0.187
	*p*-value	ns	ns	ns	ns	ns	<0.05	<0.01	<0.0001	<0.0001	<0.01
**Females**											
TyG	R squared	0.006	0.014	0.188	0.214	0.218	0.109	0.147	0.091	0.418	0.221
	*p*-value	ns	ns	<0.0001	<0.0001	<0.0001	<0.05	<0.01	ns	<0.0001	<0.0001
TyG-WC	R squared	0.213	0.235	0.722	0.721	0.945	0.660	0.311	0.320	0.428	0.239
	*p*-value	<0.0001	<0.0001	<0.0001	<0.0001	<0.0001	<0.0001	<0.0001	<0.0001	<0.0001	<0.0001
TyG-BMI	R squared	0.197	0.090	0.855	0.967	0.735	0.795	0.332	0.306	0.325	0.254
	*p*-value	<0.0001	ns	<0.0001	<0.0001	<0.0001	<0.0001	<0.0001	<0.0001	<0.0001	<0.0001

Acronyms: BW, body weight; BMI, body mass index; WC, waist circumference; HC, hips circumference; SBP, systolic blood pressure; DBP, diastolic blood pressure; MetS, metabolic syndrome; MASLD, non-alcoholic fatty liver disease; TyG, triglyceride glucose index; ns, not significant.

## Data Availability

The dataset generated and analyzed during the study is available from the corresponding author upon a reasonable request.

## Abbreviations

The following abbreviations are used in this manuscript:

MASLD	Metabolic Associated Fatty Liver Disease
MetS	Metabolic Syndrome
HDL-C	High-Density Lipoprotein Cholesterol
NASH	Non-Alcoholic Steatohepatitis
TyG	Triglyceride-Glucose Index
TyG-BMI	Tyg-Body Mass Index
TyG-WC	Tyg-Waist Circumference
BWRP	Body Weight Reduction Program
BMI	Body Mass Index
SDS	Standard Deviation Score
BW	Body Weight
WC	Waist Circumference
HC	Hip Circumference
FPG	Fasting Plasma Glucose
T-C	Total Cholesterol
TG	Triglycerides
SBP	Systolic Blood Pressure
DBP	Diastolic Blood Pressure
ROC	Receiver Operating Characteristic
AUC	Area Under the Curve
CI	Confidence Interval
PPV	Positive Predictive Value
NPV	Negative Predictive Value
PRL	Positive Likelihood Ratio
NLR	Negative Likelihood Ratio
IRCCS	Istituto di Ricovero e Cura a Carattere Scientifico
